# Mitochondrial Ribosomal Proteins and Cancer

**DOI:** 10.3390/medicina61010096

**Published:** 2025-01-09

**Authors:** Huiyi Wu, Xiaowei Zhu, Huilin Zhou, Min Sha, Jun Ye, Hong Yu

**Affiliations:** 1 Department of Pathology, The Affiliated Taizhou People’s Hospital of Nanjing Medical University, Taizhou 225300, China; wuhuiyi-6@stu.njmu.edu.cn (H.W.); zhuxiaowei20230419@njmu.edu.cn (X.Z.); zhouhuiling20230419@njmu.edu.cn (H.Z.); 2 Translational Medicine Center, The Affiliated Taizhou People’s Hospital of Nanjing Medical University, Taizhou 225300, China; shamin@njmu.edu.cn (M.S.); yejun20230206@njmu.edu.cn (J.Y.)

**Keywords:** mitochondrial dysfunction, mitoribosome, apoptosis, biomarker, precision oncology

## Abstract

Mitochondria play key roles in maintaining cell life and cell function, and their dysfunction can lead to cell damage. Mitochondrial ribosomal proteins (MRPs) are encoded by nuclear genes and are assembled within the mitochondria. MRPs are pivotal components of the mitochondrial ribosomes, which are responsible for translating 13 mitochondrial DNA-encoded proteins essential for the mitochondrial respiratory chain. Recent studies have underscored the importance of MRPs in cancer biology, revealing their altered expression patterns in various types of cancer and their potential as both prognostic biomarkers and therapeutic targets. Herein, we review the current knowledge regarding the multiple functions of MRPs in maintaining the structure of the mitochondrial ribosome and apoptosis, their implications for cancer susceptibility and progression, and the innovative strategies being developed to target MRPs and mitoribosome biogenesis in cancer therapy. This comprehensive overview aims to provide insights into the role of MRPs in cancer biology and highlight promising strategies for future precision oncology.

## 1. Introduction

The mitochondrion, a semi-autonomous organelle, possesses unique machinery for transferring information from its DNA (mtDNA) to proteins. This intricate process enables the mitochondrion to fulfill vital functions such as replication, transcription, and translation, thereby maintaining its genetic identity and supporting its pivotal role in cellular metabolism [1]. Human mitochondria contain approximately 1500 proteins, of which only 13 are encoded by mtDNA [2]. The vast majority of these proteins are encoded by nuclear DNA and are, subsequently, imported into the mitochondria. Therefore, mitochondrial genome evolution is characterized by a tight co-evolution with the nuclear genome, driven by the necessity for coordinated expression of mitochondrial and nuclear-encoded proteins essential for mitochondrial function [3,4,5]. This process is influenced by high mutation rates in mtDNA due to factors such as lack of protective histones and limited DNA repair mechanisms, leading to significant implications for aging and disease [3,6]. The dynamic interplay between nuclear and mitochondrial genomes shapes the somatic evolution of mitochondrial DNA throughout an organism’s lifetime [3].

Mitoribosomes play a pivotal role in the life activities of cells by orchestrating the synthesis and translation of 13 proteins vital for maintaining mitochondrial function and energy metabolism. This process is fundamental to the normal survival and metabolic activity of cells, ensuring their ongoing health and vitality. Human mitoribosomes are composed of a small 28S subunit and a large 39S subunit, together forming a functional 55S ribosome [7]. These mitoribosomes consist of RNA molecules and mitochondrial ribosomal proteins (MRPs), which are encoded by the mitochondrial and nuclear genomes, respectively [8]. The MRPs, encoded by nuclear genes and synthesized by the cytoplasm 80S ribosomes, contain 30 small mitoribosomal proteins (MRPSs) and 52 large mitoribosomal proteins (MRPLs) [9]. The review briefly outlines the structure of mitoribosomes and the function of MRPs, as well as their involvement in cancer.

## 2. MRPs and Mitoribosome Structure

Unlike cytoplasmic ribosomes encapsulated by a membrane, mitoribosomes lack such an encapsulation and have an irregular granular appearance [10]. In comparison to bacterial ribosomes and eukaryotic cytoplasmic ribosomes, both of which typically consist of 33% protein and 67% RNA, the ratio of MRP to RNA in mitoribosomes is notably reversed, falling approximately within the range of 25% to 30% [11,12]. Mammalian mitoribosomes have a low sedimentation coefficient of 55S, consisting of 28S small subunits (mtSSU) and 39S large subunits (mtLSU). The mtSSU is composed of 12S mitochondrial rRNA and 32 MRPs and provides a platform for mRNA binding and decoding. It is pivotal to translate leaderless mRNA, meticulously selecting translation initiation sites and exhibiting conformational adaptability during the translation process. In contrast, the mtLSU is composed of 16S mitochondrial rRNA and 50 MRPs [2]. The mtLSU plays a pivotal role in the catalytic peptidyl transferase reaction, which is essential for protein synthesis within the mitochondria. Its attributes include intricate interactions with mitochondria tRNA molecules to enable the accurate assembly of amino acids into proteins, the adaptation of the exit tunnel to ensure a smooth passage for hydrophobic nascent peptides emerging from the ribosomes, and the extensive remodeling of the central projection to facilitate the recruitment of specific tRNA molecules such as mitochondrial valine transfer RNA.

**Figure 1:**
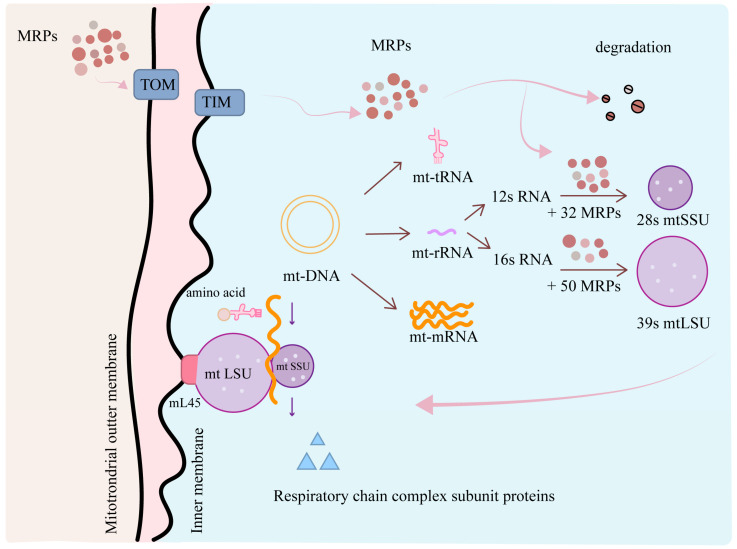
Mitoribosome structure and biogenesis.

Unlike the cytoplasmic ribosomes, mitoribosomes are, instead, anchored to the inner mitochondrial membrane through the MRPL45 of their large subunit [2]. Mitoribosome biogenesis takes place in specific compartments within the mitochondrial matrix near the mtDNA nucleoid termed RNA granule [13]. This compartment contains RNA-processing enzymes, MRPs, and other essential proteins that are pivotal for mitoribosome biogenesis (Figure 1). Given that mtDNA encodes only 13 proteins, mitochondria rely heavily on the nucleus and various cellular compartments as the primary source for the vast majority of their proteins and lipids [2]. This intricate interplay underscores the interdependence between these two genetic systems in sustaining the vital functions of mitochondria. These intricately orchestrated nuclear-encoded subunit proteins are synthesized in the cytosol and imported into the mitochondria for their subsequent assembly. The transmembrane transport process of these proteins is a highly orchestrated event, mediated by chaperones and the sophisticated transmembrane transport complex, including components such as the translocase of outer mitochondrial membrane (TOM) and the translocase of the inner membrane (TIM) [14]. In addition, these 13 proteins are indispensable for the proper functioning of the oxidative phosphorylation (OXPHOS) system [15,16]. MRPs play an indirect yet crucial role in energy metabolism by facilitating the synthesis of 13 proteins that are integral components of the electron transport chain (ETC) and the ATP synthase complex, both of which are central to the OXPHOS process [17].

Cryo-electron microscopy (cryo-EM) has indeed revolutionized structural biology by enabling the determination of macromolecular structures at a near-atomic resolution [18]. This level of detail allows researchers to visualize not only the overall architecture, but also the fine features of complex biological assemblies such as mitoribosomes [19]. By capturing high-resolution snapshots of mitoribosomes in various functional states, cryo-EM provides indispensable insights into the mechanisms underlying mitochondrial translation and offers a powerful platform for investigating the molecular basis of mitoribosome-related diseases. The mtSSU is divided into three regions, namely head, platform, and foot. The head region of the mtSSU is characterized by the presence of death-associated protein 3 (DAP3), which interacts with other ribosomal proteins and rRNA to form a stable structure [20,21,22]. It forms two bridges with MRPL46 and MRPL48 by interacting with the central protuberance (CP) of the mtLSU. The foot region of the mtSSU is characterized by the presence of the pentapeptide repetition (PPR) domain protein of MRPS27, which is thought to play a role in the recognition and binding of specific RNA molecules. The interaction between MRPS27 and the N-terminal extension of MRPL19 forms a bridge that physically links the mtSSU and mtLSU. These interactions contribute to maintain the three-dimensional conformation of the mitochondrion, which is essential for its proper functioning. The structure of the mtLSU is more complex and larger than that of the mtSSU, and it also contains both rRNA molecules and numerous ribosomal proteins. The mtLSU accommodates tRNA-binding sites, the L1 stalk, the P stalk essential for recruiting translation factors, the peptidyl transferase center (PTC) that serves as the catalyst for the formation of peptide bonds between amino acids, the CP that facilitates seamless communication among various functional sites, and the exit tunnel through which the synthesized protein is egressed. The assembly of mitoribosomes is a highly coordinated and complex process that involves an extensive network of assembly factors and GTPases. To date, there have been few studies focused on the assembly pathway of the mammalian mtSSU [23,24]. The assembly of the mtSSU encompasses factors including ERAL1, NOA1/MTG3, MCAT, METTL17, TFB1M, RBFA, METTL15, and MTIF3 [19]. In contrast, the assembly of the mtLSU involves a different set of factors, including DDX28, GTPBP10, MRM3, the MALSU1-LOR8F8-mt-ACP module, NSUN4, MTERF4, MRM2, GTPBP7/MTG1, GTPBP5/MTG2, MtEF-TW/TUFM, and GTPBP6. Although the entrance to the human mitochondrion is wider than that of bacteria due to the absence of uS4 and the deletion of the C-terminal domain of uS3, the diameter of the mRNA channel is still smaller than that of an RNA duplex, thereby allowing only single-stranded mRNA to enter [21].

## 3. MRPs and Apoptosis

In addition to their role in energy production, mitochondria play a crucial role in apoptosis or programmed cell death. Alterations in mitochondrial function and defects in apoptosis are well-recognized hallmarks of cancer cells [25]. Dysregulation of MRPs can lead to mitochondrial dysfunction, which disrupts cellular energy metabolism and the balance of pro- and anti-apoptotic factors, thereby contributing to the survival and proliferation of cancer cells [17,26]. Specifically, MRPs such as DAP3, MRPL41, and MRPS30 are involved in the regulation of apoptosis.

**Figure 2:**
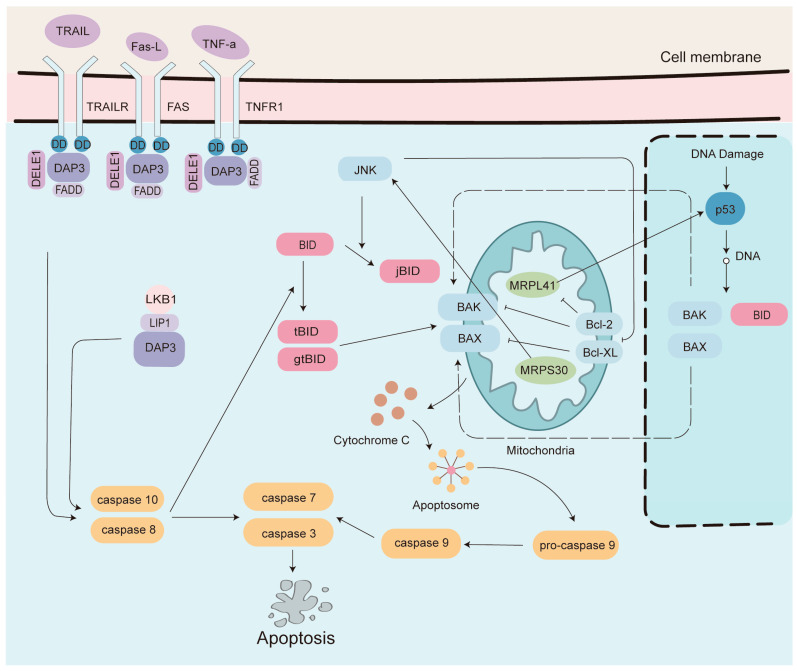
The roles of DAP3, MRPL41, and MRPS30 in apoptosis.

DAP3 is a multifunctional protein that not only contributes to the assembly and function of the mtSSU, but also acts as a pro-apoptotic factor by interacting with other proteins to promote cell death [27]. DAP3 actively participates in the extrinsic apoptotic pathway, triggered by the tumor necrosis factor (TNF), the Fas ligand, and the TNF-related apoptosis-inducing ligand (TRAIL) [28,29]. As shown in Figure 2, DAP3 directly binds to the death domains (DD) of death receptors (DRs), including TNF receptor type 1 and TRAIL receptors DR4 and DR5 [27,30]. This binding enables DAP3 to form a complex with caspase-8 through Fas-associated death domain proteins, thereby initiating the apoptotic process. DAP3 also interacts with DAP3 binding cell death enhancer 1 (DELE1) to coordinate apoptotic events [30]. Notably, DELE1 knockdown enhances cell viability and inhibits DR-mediated apoptosis in response to the stimulation of TNF, anti-Fas, or TRAIL. Furthermore, liver kinase B1 (LKB1) has been shown to facilitate TRAIL-mediated apoptosis through the interaction of LKB1 interacting protein 1 with DAP3 [31]. This suggests a role for LKB1 in enhancing the sensitivity of cells to TRAIL-induced apoptosis through this specific protein–protein interaction network. Moreover, DAP3 is a key player in anoikis, the detachment-induced apoptosis of epithelial cells, through its interaction with the FAK-caspase-8 axis [32]. The regulation of the pro-apoptotic function of DAP3 is complex, modulated by various factors including AKT/PKB-mediated phosphorylation, transcriptional regulation of HML-10 transcripts, and post-transcriptional regulation by miR-365-1 [33,34,35]. These various regulatory mechanisms highlight the intricate balance that governs the function of DAP3 in cellular processes, particularly in the context of apoptosis.

MRPL41, also known as Bcl-2 interacting mitochondrial ribosomal protein (BMRP), is a component of the mtLSU. MRPL41 has the ability to trigger apoptosis through a p53-dependent pathway, with Bcl-2 and caspases serving as key mediators [36]. The 13–17 amino acid region of MRPL41 is necessary for binding to Bcl-2, with aspartic acid residue 16 being essential for this interaction. In addition, MRPL41 enhances the stability of p53 and facilitates its translocation to the mitochondria, thereby inducing p53-dependent apoptosis. The apoptosis-inducing ability of MRPL41 can be inhibited by Bcl-2 or by the caspase inhibitor. A recent study by Guo et al. [37] reported that inhibition of MRPL41 enhanced cell viability and increased Bcl-2 expression, in addition to suppressing apoptosis and downregulating p53. These multifaceted effects of MRPL41 underscore its significant role in regulating cell survival and cell death pathways. MRPS30, also known as programmed cell death protein 9 (PCDP9), is a component of the mtSSU. Although MRPS30 has also been implicated in the regulation of apoptosis through the activation of c-Jun N-terminal kinase 1 (JNK1) in murine fibroblasts [38], the exact mechanisms by which MRPS30 contributes to apoptosis are not as well characterized as those of MRPL41.

## 4. MRPs Associated with Cancers

Cancer is a complex disease characterized by its diverse manifestations and underlying causes, resulting from perturbations in multiple cellular processes. These alterations include the disruption of growth signals, the evasion of apoptosis, unlimited replication potential, angiogenesis, invasion, and metastasis [25]. In 1926, Otto Warburg first described the differences in the glycolytic production of ATP under aerobic conditions between tumor cells and normal cells, a phenomenon now known as the Warburg effect [39]. Mitochondrial ribosomes play a crucial role in mitochondrial function. In recent years, numerous studies have uncovered potential mechanisms by which MRPs are involved in tumorigenesis. MRPs are closely linked to the occurrence and development of cancer. Therefore, the characterization and understanding of dysregulated MRPs in cancer may provide a powerful tool for cancer diagnosis, prognosis, and treatment outcomes.

### 4.1. MRPs and Lung Cancer

**Figure 3:**
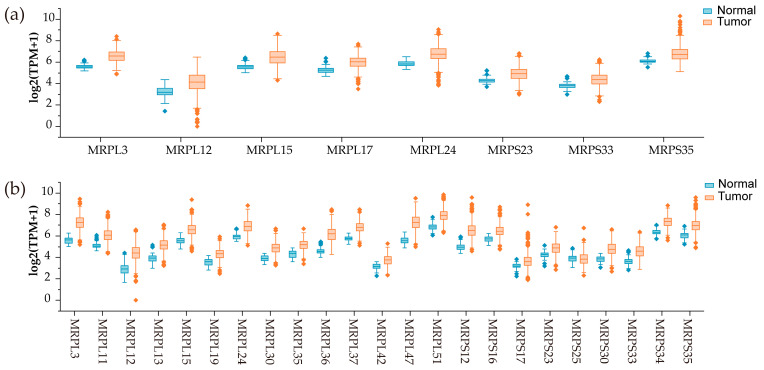
Differentially expressed MRPs in relation to lung cancer. (**a**) Eight upregulated differentially expressed MRPs in LUADs; (**b**) 22 upregulated and one downregulated differentially expressed MRPs in LUSCs. |log2 (FC)|> 1, q value < 0.01.

Lung cancer is the most common type of cancer and the primary cause of cancer-related deaths globally [40]. Given that metabolic reprogramming is a key feature of lung cancer, the dysregulation of MRPs can lead to altered energy metabolism, thereby supporting the rapid growth and survival of cancer cells [17,41]. Bennett et al. [42] found that silencing mitochondrial ribosomal proteins and respiratory chain genes in primary and metastatic lung cancers lead to significant changes in mitochondrial respiration, with primary tumors showing a greater dependence on these genes for growth compared to metastatic tumors. This suggests that primary tumors are more sensitive to disruptions in mitochondrial function, whereas metastatic tumors may have developed alternative metabolic pathways. The energy metabolic phenotypes of lung cancer are not singular but rather represent a spectrum of metabolic states that evolve as the disease progresses [42,43]. In addition, the gene expression profiling interactive analysis (GEPIA) database reveals differential expression of MRPs in lung cancers [44]. Specifically, eight MRPs are significantly upregulated in lung adenocarcinomas (LUADs), while, in lung squamous cell carcinomas (LUSCs), 22 MRPs are upregulated and 1 MRP is downregulated (Figure 3). Notably, seven MRPs (MRPL12, MRPL3, MRPL15, MRPL24, MRPS23, MRPS33, and MRPS35) have been found to be all upregulated in both LUADs and LUSCs. This indicates that these shared MRPs may play a fundamental role in mitochondrial function across different subtypes of lung cancer, potentially indicating common metabolic adaptations or vulnerabilities that could be targeted therapeutically.

MRPL12 is a key player in mitoribosome biogenesis and transcription, interacting with mitochondrial RNA polymerase to activate mtDNA transcription and regulate gene expression [45]. Therefore, MRPL12 is essential for maintaining mitochondrial homeostasis, particularly in the regulation of mitochondrial respiration [46]. MRPL12 knockdown induces structural damage in mitochondria, characterized by swelling, irregular arrangement, less recognizable cristae, and reduced volume and abundance. MRPL12 is upregulated in many types of cancer, including lung cancer, hepatocellular carcinoma (HCC), and breast cancer [46,47]. MRPL12 enhances cell proliferation, migration, and invasion of lung cancer cells by upregulating mitochondrial oxidative phosphorylation, and its expression levels are inversely associated with the infiltration levels of multiple immune cells. Similar findings have also been observed in HCC, where MRPL12 promotes tumor growth and metastasis through the regulation of mitochondrial metabolism [45]. High expression levels of both MRPL12 mRNA and protein are associated with a poor prognosis in lung cancer as well as HCC [45,47]. The MRPL12 Y60 site is a specific phosphorylation modification site that regulates its oncogenic functions [46]. Furthermore, Liu et al. [48] observed an oncogenic role of MRPL12 that promoted cell proliferation and migration of breast cancer cells. High MRPL12 expression was weakly but significantly associated with a poor prognosis in breast cancer. These results indicate that MRPL12 may be a promising prognostic biomarker and therapeutic target for lung cancer, breast cancer, and HCC.

MRPL13 has been involved in various biological processes, including participation in protein synthesis within the mitochondrion, the adaptation of the translation system to the specific requirements of the organelle, and the structural and functional integrity of the mitochondrion [49,50]. Furthermore, many studies have demonstrated that MRPL13 has a significant role in cancer. MRPL13 is dysregulated in many types of cancer, including breast cancer, colorectal cancer (CRC), gastric cancer, and lung cancer [51,52]. High MRPL13 expression is associated with a worse prognosis in several types of cancer, including lung cancer, breast cancer, and gastric cancer [51,52,53,54]. There is an inverse association between MRPL13 expression and the abundance of tumor-infiltrating lymphocytes (TILs), such as B cells, CD8+ T cells, and macrophages [51]. MRPL13 expression influences the sensitivity to anticancer drugs, including tramadol, CI-1040, navitoclax, and nutlin-3A. Zhong et al. [51] observed that MRPL13 knockdown promoted the apoptosis and inhibited division, metastasis, and invasion in LUADs. Cai et al. [53] reported that MRPL13 promoted breast cancer invasion, metastasis, and epithelial–mesenchymal transition (EMT) through the PI3K/AKT/mTOR signaling pathway, highlighting its involvement in cancer progression. High MRPL13 expression was associated with adverse clinicopathological variables and unfavorable clinical outcomes in breast cancer patients. A recent study by Dai et al. [54] also demonstrated that MRPL13 facilitated the invasion of breast cancer cells. The expression levels of MRPL13 in metastatic breast cancer tissues were obviously higher than those in primary breast cancer tissues. Wang et al. [52] observed that MRPL13 promoted cell proliferation and G1/S phase transition in gastric cancer cells and enhanced xenograft growth by suppressing the p53 signaling pathway. Lee et al. [55] reported that exogenous lactate inhibited mitochondrial OXPHOS by downregulating MRPs, particularly MRPL13, thereby facilitating invasion of HCC cells through the induction of claudin-1. Taken together, MRPL13 is a diagnostic and prognostic biomarker for several types of cancer, including lung cancer, breast cancer, and gastric cancer [51,53,54].

MRPL9 is a component of the mtLSU and is upregulated in many types of cancer, including lung cancer, HCC, and breast cancer [56]. Li et at. [57] observed that MRPL9 promoted cell proliferation, migration, and invasion of lung cancer cells by regulating c-MYC and its overexpression was associated with a poor prognosis in lung cancer patients, indicating that MRPL9 may be a potential therapeutic target for lung cancer. Upregulation of MRPL15 is observed in many types of cancer, including lung cancer, CRC, and gastric cancer [58]. The expression levels of MRPL15 are inversely correlated with the infiltration levels of multiple immune cells. High expression of both MRPL15 mRNA and protein is associated with a poor prognosis in lung cancer patients. Similarly to MRPL15, upregulation of MRPL19 is observed in many types of cancer, including lung cancer, CRC, and gastric cancer [59]. Wei et al. [59] found that MRPL19 knockdown induced apoptosis and inhibited the migration and invasion of lung cancer cells. The expression levels of MRPL19 were inversely correlated with the infiltration levels of B cells, CD4+ T cells, and dendritic cells. High expression of both MRPL19 mRNA and protein were associated with a poor prognosis in lung cancer patients. MRPL51 is crucial for mitochondrial genome stability, with a key role in redox homeostasis [60]. Lack of MRPL51 leads to loss of mitochondrial genome and impaired oxidative phosphorylation, reducing superoxide production. A recent study by Zhang et al. [61] reported that MRPL51 was transcriptionally activated by FOXM1 and its mRNA, and that protein levels were upregulated in LUADs. At the single-cell level, the expression level of MRPL51 exhibited a positive correlation with various cellular processes in LUAD cells, including the cell cycle, DNA damage, DNA repair, EMT, invasion, and proliferation. Upregulation of MRPL51 contributed to the malignant behaviors of LUAD cells and was associated with a poor prognosis. Gu et al. [62] reported a risk model based on three differentially methylated genes, including MRPL51, SLK, and PRC1, which exhibited promise for guiding the management of LUAD treatment. Guttapadu et al. [63] found that MRPL52 was upregulated in LUAD and LUSC patients, and its overexpression was correlated with a poor prognosis in LUSC patients.

### 4.2. MRPs and Breast Cancer

Breast cancer is the most commonly diagnosed cancer and the fifth leading cause of cancer-related deaths among women in China [64]. Although MRPs are dysregulated in breast cancer patients, including the aforementioned MRPL9, MRPL12, and MRPL13, the specific mechanisms underlying the activity of these proteins in relation to this cancer have yet to be explored.

Lin et al. [65] analyzed the expression patterns and prognostic significance of MRPs in breast cancer patients using the GEPIA database. They identified 12 upregulated MRPs, including MRPL3, MRPL13, MRPL14, MRPL17, MRPL24, MRPL42, MRPL47, MRPS23, DAP3, MRPS30, MRPS34, and MRPS35. Notably, MRPL13, MRPL14, MRPL17, MRPL42, DAP3, and MRPS35 were upregulated across all four subtypes. A prognostic model including four MRPs (MRPL16, MRPL40, MRPS18C, and MRPS35) demonstrated a good performance in predicting the survival outcomes of breast cancer patients. Furthermore, we conducted an analysis of 1,113 breast cancer samples from The Cancer Genome Atlas (TCGA) to identify differentially expressed MRPs across various subtypes. Our findings revealed distinct expression patterns of MRPs that varied significantly between different breast cancer subtypes (Table 1). These expression patterns were inconsistent with the results reported by Lin et al. [65], something which may be attributable to differences in sample size. Five MRPs (MRPL12, MRPS12, MRPL13, MRPL14, and MRPS34) were upregulated across all five subtypes. There was subtype-specific upregulation of MRPs, including MRPL17, MRPS23, MRPL21, and MRPS28 in the luminal B subtype, and GADD45GIP1, MRPL2, MRPL4, MRPL9, MRPS21, MRPL36, MRPL37, and MRPL51 in the basal-like subtype. These subtype-specific patterns suggest potential oncogenic influences on cancer progression, highlighting the diverse roles of MRPs in different breast cancer subtypes.

MRPS23 is upregulated in various types of cancer, including breast cancer, HCC, pancreatic adenocarcinoma, and glioma [66]. The aberrant expression of MRPS23 is correlated with a poor prognosis in relation to many types of cancer, including breast cancer [66,67]. Although Gatza et al. [68] observed an association of MRPS23 amplification with poor prognosis in breast cancer patients, this finding was not validated by Klæstad et al. [69]. Two studies reported that MRPS23 knockdown in breast cancer cells suppressed proliferation, metastasis, and invasion while promoting apoptosis and increasing sensitivity to paclitaxel [70,71]. Furthermore, the phosphorylation of MRPS23 by mitotic kinases influenced cell proliferation, migration, and enhanced sensitivity to CDK1 inhibitors in breast cancer cells [67,72]. Liu et al. [67] found that methylation of arginine 21 and lysine 108 on MRPS23 coordinatively promoted breast cancer cell survival and malignancy by inhibiting OXPHOS. These findings suggest that the role of MRPS23 in breast cancer may be more complex and nuanced than initially thought. Furthermore, MRPS23 also contributes to the malignant phenotypes of other types of cancer, including glioma, HCC, and cervical cancer [66,73,74]. These findings indicate that MRPS23 may play a significant role in promoting the aggressive traits of these cancers. Further studies are required to fully elucidate its exact mechanisms in these diseases.

MRPS27 functions as an RNA-binding protein that interacts with 12S mitochondrial rRNA and participates in the translation of mRNAs [75]. A recent study by Zheng et al. [76] found that MRPS27 was upregulated in triple-negative breast cancer (TNBC). MRPS27 promoted cell proliferation, migration, invasiveness, and tumor growth in TNBC. Inhibition of MRPS27 triggered nucleolar stress and suppressed the stemness properties in TNBC. Upregulation of MRPS27 diminished the stemness-inhibitory effects of lovastatin in TNBC cells, indicating an important role of MRPS27 in maintaining the stemness properties. High MRPS27 expression was associated with advanced TNM stage and a worse prognosis in TNBC patients. Further studies are required to explore the mechanisms of MRPS27 and validate its prognostic significance in the context of breast cancer. MRPL52 is upregulated in breast cancer, particularly in metastatic cases [8]. Breast cancer patients with high MRPL52 expression have a poor prognosis. MRPL52 regulates hypoxia-induced apoptotic resistance and metastatic initiation of breast cancer cells by facilitating PINK1/Parkin-dependent mitophagy and transactivating the reactive oxygen species (ROS)-Notch1-Snail pathway, respectively.

Neoadjuvant chemotherapy (NAC) is the standard of care for locally advanced breast cancer and increases the rate of breast-conserving surgery. Vishnubalaji et al. [77] identified a gene signature including MRPL9 and MRPL37 associated with resistance to NAC in TNBC at the single-cell level. MRPL9 and MRPL37 were upregulated in TNBC resistant to NAC. Inhibiting MRPL37 lead to a reduction in TNBC colony formation and an enhancement in sensitivity to paclitaxel, but these effects were not observed when MRPL9 was inhibited. To date, there are limited reports on MRPL9 and MRPL37 in cancer, and our understanding of their roles in the disease is still limited.

Previous studies have demonstrated that the 5p12 variants rs10941679 and rs7716600 confer susceptibility to estrogen receptor (ER)-positive breast cancer [78,79,80]. The rs10941679 is located within an enhancer element that physically interacts with the promoter regions of FGF10 and MRPS30 [79]. Although there is no difference in transcription factor binding affinity between rs10941679 alleles, the G allele confers a higher expression of FGF10 and MRPS30. Furthermore, rs7716600 is the variant in the 5p12 region with the greatest effect on the expression level of MRPS30, and the risk A allele is associated with decreased methylation at the MRPS30 promoter site in ER-positive breast cancer cells [80]. It is interesting that MRPS30 is not expressed in normal breast luminal epithelial cells but is significantly upregulated in breast cancer patients, a phenomenon which contradicts its role in promoting apoptosis [38,81,82]. MRPS30 is dysregulated in many types of cancer, including breast cancer, CRC, lung cancer, and gastric cancer [17]. The mechanism of MRPS30 in the development and progression of ER-positive breast cancer is still not clearly understood.

### 4.3. MRPs and Digestive System Cancers

**Figure 4:**
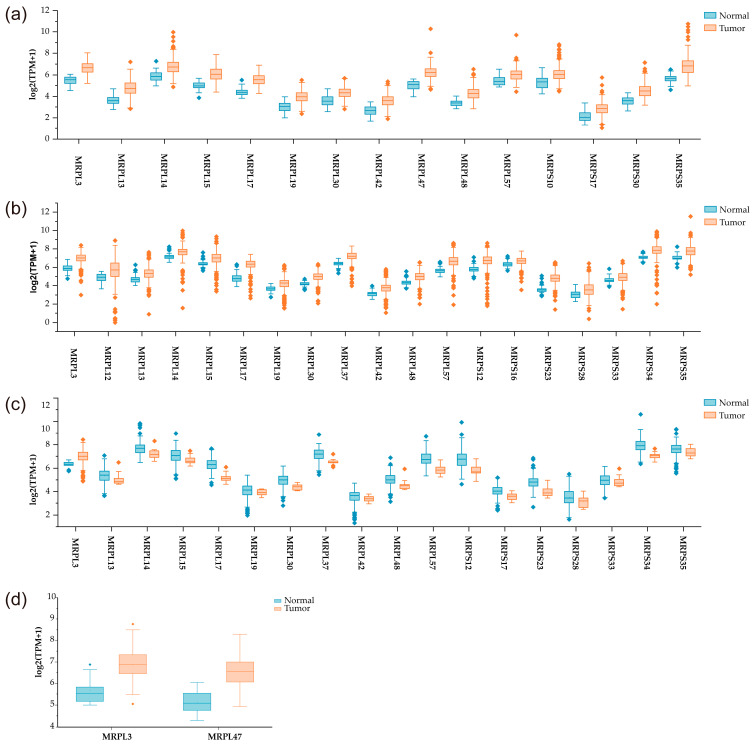
Differentially expressed MRPs in digestive system cancers. (**a**) Gastric cancer; (**b**) COAD; (**c**) READ; (**d**) ESCA. |log2 (FC)| > 1, q value < 0.01.

Gastric cancer is a prevalent malignant tumor of the gastrointestinal tract, ranking fifth for incidence and fourth for mortality, globally [40]. According to the GEPIA database, 16 MRPs are significantly upregulated in gastric cancer (Figure 4) [44]. These MRPs include MRPL3, MRPL13, MRPL14, MRPL15, MRPL17, MRPL19, MRPL30, MRPL42, MRPL47, MRPL48, MRPL57, MRPS30, MRPS10, MRPS17, and MRPS35. The significant upregulation of these MRPs indicates their potential involvement in altered mitochondrial function and metabolism in gastric cancer cells. MRPS17 is upregulated in many types of cancer, including gastric cancer [83,84,85]. Upregulated MRPS17, along with the elevated expression of other MRPs, enhances OXPHOS in cancer cells, thereby fueling cancer metabolism [86]. Zhou et al. [83] reported that MRPS17 facilitated the migration and invasion of gastric cancer cells by regulating the PI3K/AKT signaling pathway. High MRPS17 expression was associated with a worse prognosis in gastric cancer patients. Li et al. [87] developed a seven-gene signature including MRPS17 for predicting the prognosis of patients with gastric cancer. The expression level of MRPS17 was inversely correlated with the abundance of TILs, including Th1 cell, Tem-CD8 cell, NKT cell, and NK cell. A recent study by Hu et al. [88] identified a three-gene signature including MRPS17, GUF1, and PDK4 for predicting the prognosis of gastric cancer patients. These findings imply that MRPS17 may serve as a prognostic biomarker for gastric cancer. MRPL35 is involved in the formation of the CP of the mtLSU, and its interaction with other MRPs such as MRPL38 is essential for optimizing the mitotranslational process to ensure the synthesis of competent proteins for the assembly into functional OXPHOS enzymes, particularly cytochrome c oxidase [89,90]. MRPL35 is upregulated in several types of cancer, including lung cancer, CRC, gastric cancer, and HCC, and its overexpression is associated with a worse prognosis in these cancer types [91,92,93,94,95]. High MRPL35 expression is correlated with an advanced stage of gastric cancer [92]. MRPL35 knockdown induces apoptosis in gastric cancer cells, inhibits their proliferation and colony formation, and suppresses tumor growth by regulating BCL-XL, PICK1, and AGR2. 18β-glycyrrhetinic acid (18β-GRA), extracted from the natural medicine licorice, has demonstrated reliable anticancer effects on human cancer cells [96]. 18-GRA inhibits cell proliferation and tumor growth and promotes apoptosis by downregulating MRPL35 in gastric cancer patients. MRPL35 knockdown inhibits cell proliferation and tumor growth in CRC by triggering the accumulation of ROS, which subsequently leads to DNA damage, thereby suppressing the disease progression in CRC [91]. There is a significantly lower level of the promoter methylation of MRPL35 in HCC, a phenomenon which is consistent with its upregulation in HCC [95]. MRPL35 promotes migration and invasion of HCC cells, confers resistance to anticancer drugs, and likely exerts negative effects on the immune system. Wang et al. [97] reported that MRPL35 knockdown inhibited cell proliferation and induced apoptosis in esophageal carcinoma (ESCA) TE-1 cells. In addition, MRPL35 facilitates cell proliferation, invasion, glutamine metabolism, and tumor growth by regulating SLC7A5 in lung cancer patients, whereas it is deubiquitinated and stabilized by ubiquitin-specific protease 39 [93]. MRPL35 may play important roles in cancer progression and confer multidrug resistance, particularly in relation to gastric cancer and lung cancer. Sotgia et al. [98] identified 12 MRPLs and eight MRPSs that showed a significant prognostic value and developed an eight-gene signature including MRPL28 and MRPL52 for predicting the prognosis in gastric cancer patients. A more recent study by Wang et al. [99] reported that a nine-gene signature including MRPL4 served as an independent indicator for the prognosis in gastric cancer patients and was associated with immune escape mechanisms as well as the response to PD-L1 immunotherapy. Further research into the specific mechanisms by which these MRPs contribute to cancer progression could provide valuable insights and lead to the identification of novel therapeutic strategies targeting mitochondrial pathways.

HCC is the fourth most common cancer and the second leading cause of cancer-related deaths in China [64]. Many MRBs are associated with the progression of HCC, including the aforementioned MRPL9, MRPL12, MRPL13, MRPS23, and MRPL35. A recent study by Zhao et al. [100] identified 14 differentially expressed MRPs (MRPS17, MRPS21, MRPS23, MRPL9, DAP3, MRPL13, MRPL14, MRPL15, MRPL16, MRPL17, MRPL21, MRPL24, MRPL47, and MRPL55) in HCC from TCGA, all of which exhibited a good diagnostic performance. Furthermore, the study also revealed that 39 MRPs were associated with a better or worse prognosis in HCC. Previous studies have demonstrated that MRPL21 may potentially play a role in several critical cellular processes, including the activation of the cell cycle, the enhancement of apoptotic pathways, and the triggering of DNA damage responses [100]. These multifaceted functions highlight the importance of MRPL21 in maintaining cellular homeostasis and responding to stress. MRPL21 is dysregulated in some types of cancer, including HCC and ESCA [100,101,102]. The aberrant expression of MRPL21 is associated with a poor prognosis of HCC and with the drug resistance of acute myeloid leukemia (AML) and LUSC [101,103,104]. Upregulation of MRPL21 promotes cell proliferation and confers resistance to the apoptosis of HCC cells, which may be responsive because of the increased incidence of TP53 mutations [101]. Nutlin-3, a P53 inhibitor, effectively potentiates the pro-apoptotic effects of MRPL21 knockdown in HCC cells. These findings highlight the importance of MRPL21 in the progression of HCC and its potential as a prognostic biomarker and therapeutic target. MRPL48 is upregulated in many types of cancer, including HCC, breast cancer, and gastric cancer, a phenomenon which may be due to the hypomethylation of its promoter [105]. Dysregulation of MRPL48 has been observed in HBV-, non-alcoholic steatohepatitis-related HCC, but not in HCV-related HCC. MRPL48 promotes the cell proliferation, migration, and invasion of HCC cells and influences immune infiltration levels in HCC. High MRPL48 expression is associated with a more aggressive behavior of HCC and a worse prognosis. Hu et al. [106] reported that MRPL48 knockdown inhibited cell proliferation and enhanced cetuximab sensitivity in CRC. However, there is limited research and understanding regarding the role of MRPL48 in cancer, and its role in cancer is not well understood. Further studies are required to elucidate the mechanisms by which MRPL48 contributes to cancer development and progression.

CRC is the second most common cancer and the fourth leading cause of cancer-related deaths in China [64]. Previous studies have revealed that some MRPs are dysregulation in CRC, including the aforementioned MRPL9, MRPL19, MRPL13, MRPL15, MRPL35, and MRPL48. Building on this foundation, our analysis using the GEPIA database identified 19 MRPs as being significantly upregulated in colon adenocarcinomas (COADs), including MRPL3, MRPL12, MRPL13, MRPL14, MRPL15, MRPL17, MRPL19, MRPL30, MRPL37, MRPL42, MRPL48, MRPL57, MRPS12, MRPS16, MRPS23, MRPS28, MRPS33, MRPS34, and MRPS35 (Figure 4) [44]. Similarly, 18 MRPs are significantly upregulated in rectum adenocarcinomas (READs), including MRPL3, MRPL13, MRPL14, MRPL15, MRPL17, MRPL19, MRPL30, MRPL37, MRPL42, MRPL48, MRPL57, MRPS12, MRPS17, MRPS23, MRPS28, MRPS33, MRPS34, and MRPS35. The alternative splicing of MRPL33 pre-mRNA is regulated by hnRNPK, leading to long (L) and short (S) isoforms [107]. The MRPL33-L abundance in cancer tissues is increased compared to that in normal tissues, including CRC and gastric cancer [107,108]. MRPL33-L exhibits an oncogenic function that facilitates cell proliferation and inhibits apoptosis in CRC, whereas MRPL33-S does not affect these phenotypes [107]. Furthermore, Li et al. [108] found that MRPL33-L and MRPL33-S play distinct roles in the response of gastric cancer cells to chemotherapy. Specifically, MRPL33-L promoted resistance to epirubicin, while MRPL33-S enhanced sensitivity to epirubicin through the PI3K/AKT signaling pathway. MRPL43 is weakly but significantly upregulated in CRC [109]. MRPL43 knockdown inhibits cell proliferation, invasion, and migration, and promotes apoptosis in CRC. Abdul Aziz et al. [110] developed a 19-gene signature including MRPL52 that was an independent predictor of survival of CRC patients. MRPL41 is dysregulated in many types of cancer, including CRC, ESCA, and lung cancer [17]. Xin et al. [111] established a nine-gene signature including MRPL41 for the prediction of postoperative recurrence in stage II/III CRC.

To date, there are very few studies specifically investigating the role of MRPs in pancreatic adenocarcinomas. Chen et al. [112] developed a four-gene signature including DNAH10, HSBP1L1, KIAA0513, and MRPL3 for predicting the prognosis of pancreatic adenocarcinoma patients from The Cancer Genome Atlas (TCGA) dataset. Patients with a high-risk score based on the signature experienced a significantly shorter overall survival time. To validate these findings, the prognostic model was tested using an independent database, confirming its robustness and predictive accuracy. The inclusion of MRPL3 in the prognostic model underscores the potential importance of MRPs in pancreatic adenocarcinomas. Interestingly, the GEPIA database has also revealed that 63 MRPs, including MRPL3, are significantly upregulated in pancreatic adenocarcinomas (Table 2) [44]. This extensive list of upregulated MRPs suggests that these proteins might play critical roles in the disease’s progression and highlights the need for further investigation into their specific contributions. Such research could lead to the identification of novel biomarkers or therapeutic targets in pancreatic adenocarcinomas.

MRPL37 is dysregulated in some types of cancer, including ESCA, CRC, and breast cancer [113]. Liu et al. [113] developed a six-gene signature including MRPL37 that was an independent indicator for ESCA. The signature was also associated with resistance to chemoradiotherapy by modulating the immune microenvironment and impacting angiogenesis. Notably, only MRPL3 and MRPL47 are upregulated in ESCA according to the GEPIA database (Figure 4) [44]. Tian et al. [114] found that MRPS18A was upregulated in cholangiocarcinomas and that its overexpression was associated with a worse prognosis. A recent study by Kim et al. [115] report that upregulated MRPS18A was associated with an improved prognosis in patients with mismatch repair-proficient advanced refractory CRC who were treated with regorafenib and nivolumab. Moreover, increased expression of MRPS18A has also been observed in HCC and breast cancer patients [116,117]. According to the GEPIA database, MRPS18A exhibits dysregulation in pancreatic adenocarcinomas, ovarian serous cystadenocarcinomas, and AML (Table 2) [44]. Zhuang et al. [118] reported that MRPL27 was upregulated in cholangiocarcinomas and that it served as an independent prognostic biomarker. Huang et al. [119] identified a four-gene signature including SDHAF2, MRPS34, MRPL11, and COX8A that served as an independent indicator for patients with cholangiocarcinomas treated with adjuvant transarterial chemoembolization. These four key genes are critical for maintaining the stemness of cholangiocarcinoma stem cells and may confer chemoresistance to cholangiocarcinoma cells.

Abnormal expression of different MRPs in the same cancer type or of specific MRPs across different cancers can significantly impact cancer behavior and progression. However, it remains unclear whether there are differences in the mechanisms by which these MRPs influence cancer behavior.

### 4.4. MRPs and Other Cancers

In addition to the aforementioned breast cancer, lung cancer, and digestive tract cancers, there are other malignancies associated with MRPs. MRPL9 is upregulated and promotes cell proliferation, metastasis, and growth by regulating the MAPK/ERK signaling pathway in papillary thyroid cancers (PTCs) [120]. The interaction between γ-glutamylcyclotransferase and MRPL9 enhances the oncogenic function of MRPL9. PTC patients with elevated MRPL9 expression tend to have a worse prognosis. MRPL15 is upregulated in ovarian cancer, a phenomenon which may be partially due to the amplification and hypomethylation of MRPL15 [121]. MRPL15 influences the abundance of infiltrating immune cells, such as CD8+ T cells and activated dendritic cells. High MRPL15 expression is associated with an advanced stage and a poor prognosis in ovarian cancer patients. MRPL51 and MRPL52 are upregulated in ovarian cancer patients, with the low expression of both MRPL51 and MRPL52 associated with a worse prognosis [122]. Moreover, MRPL51 and MRPL52 influence the abundance of infiltrating immune cells, such as γδ T and memory B cells. The transcription of MRPL52 is regulated by LINC00152 and is found to be upregulated in oral squamous cell carcinomas (OSCCs) [123]. MRPL52 promotes OSCC cells proliferation, and its overexpression is associated with a worse prognosis [123]. Gou et al. [124] found that MRPL33 was upregulated in HPV-related OSCCs, and its expression level was further increased when treated with the bromodomain inhibitor, JQ1. Wei et al. [125] identified 19 differentially expressed MRPSs (|log2 (FC)| > 1, *p* < 0.05.) out of 25 MRPSs analyzed in clear cell renal cell carcinomas (ccRCCs). Eight MRPSs (MRPS14, MRPS15, MRPS26, MRPS28, MRPS30, MRPS31, MRPS35, MRPS36) were associated with overall survival. They subsequently developed a six-MRPS signature (MRPS14, MRPS15, MRPS26, MRPS28, MRPS30, and MRPS36) for predicting the prognosis of ccRCC patients. Furthermore, two potential therapeutic targets and eight agents were identified that might enhance the therapeutic effects on patients with high MRPS-related scores. A recent study by Yang et al. [126] found that MRPS5 was significantly upregulated in ccRCC patients. However, high MRPS5 expression was associated with a better prognosis in ccRCC patients, contrasting sharply with the poor prognosis observed in HCC patients with high MRPS5 expression. This discrepancy suggests the context-dependent roles of MRPS5 across different cancer types and underscores the need for further research to understand its mechanisms in various oncological settings. In addition, further studies are required to validate the prognosis significance of MRPS5 in ccRCC patients.

Hu et al. [127] established a six-gene signature including MRPL47 for predicting the prognosis of patients with squamous cell carcinomas of the head and neck. MRPL47 rs10513762 is a potential risk factor for vincristine-induced peripheral neuropathy in childhood AML [128]. A recent study by Liu et al. [129] reported that the elevated expression of both MRPL4 and MRPS12 was significantly associated with unfavorable clinical outcomes in AML patients. Fu et al. [130] found that MRPL4 was upregulated in high-risk prostate cancer patients, and patients with elevated MRPL4 expression had a worse prognosis. MRPS23 exhibits oncogenic functions in glioma patients, promoting the cell proliferation and metastasis of glioma cells, and serves as an independent prognostic indicator [66]. Upregulation of MRPS17 in glioblastomas confers resistance to chemotherapy including temozolomide and nitrosoureas [131]. Liu et al. [132] found that MRPS7 was upregulated in osteosarcoma patients and that it might be associated with metastasis. Bell et al. [133] reported that neuroblastoma patients with elevated MRPL11 expression had a worse prognosis. Bae et al. [134] found that MRPS18B rs148828689 was significantly associated with the risk of neuroblastoma in Korean children. According to the GEPIA database, MRPS18B is dysregulated in lower-grade gliomas and AML (Table 2) [44]. Furthermore, a previous study has demonstrated that MRPS18B interacts with p53, indicating its potential role in p53-related pathways [135]. These findings indicate that MRPS18B might play important roles in cancer development and progression. Song et al. [136] found that MRPS18A gene fusions were more commonly detected in high-grade endometrial stromal sarcomas (MRPS18A-ZAN) and uterine leiomyosarcomas (MRPS18A-PDC-AS1), suggesting their potential as biomarkers for the differential diagnosis between these two types of uterine sarcomas. However, the evidence regarding the effect of other MRPs on different types of cancer is limited, warranting further investigation in the future.

Furthermore, the GEPIA database has revealed that these types of cancer exhibit distinct expression patterns of MRPs (Table 2) [44]. The vast majority of MRPs are upregulated across various cancer types, while a few MRPs show downregulation in relation to specific cancers. This widespread upregulation suggests an increased metabolic activity in cancer cells, often associated with enhanced energy production to support rapid cell proliferation. Interestingly, 59 abnormally expressed MRPs in AML patients are downregulated, a phenomenon which is a unique pattern compared to other cancers. This suggests that the dysregulation of MRPs in AML patients may involve a different set of mechanisms compared to other cancers. Additionally, MRPL1 has been observed to be only dysregulated in pancreatic adenocarcinoma patients, and other MRPs are dysregulated in relation to various types of cancer described in the article. This indicates that MRPs play critical roles in multiple cancer types and highlights the complexity of their involvement in oncogenesis.

## 5. MRPs in Cancer Therapy

Drug resistance significantly limits the effectiveness of cancer treatments and poses a major challenge to precision medicine. Numerous studies have demonstrated that the biosynthesis and translation of mitochondrial ribosomes are intricately related to tumor development, metabolism, and chemotherapy resistance [10,12,26,137,138,139]. In this context, exploring the roles of MRPs in cancer patients offers promising potential for enhancing early diagnosis, improving prognosis, and developing innovative therapeutic strategies.

Accumulating evidence indicates that MRPs can impact multiple types of cancer, with some MRPs showing abnormal expression in relation to some specific types of cancer [17,100,125]. Therefore, MRPs have the potential to serve as therapeutic targets and diagnostic and prognostic biomarkers for various cancers. As mentioned above, certain MRPs, including MRPL33, MRPL37, MRPL48, and MRPS23, have been observed to confer resistance to anticancer drugs at the cellular level [72,77,95,106]. The expression levels of MRPL21 and MRPL35 are related to resistance to anticancer drugs [95,103,104]. MRPS17 and several gene signatures containing MRPs such as MRPL37, MRPS34, and MRPL11 may be independent indicators for cancer patients treated with chemotherapy or chemoradiotherapy [113,119,131]. In addition, JG-98, an allosteric inhibitor of the mitochondrial heat shock protein 70, re-sensitizes castration-resistant prostate cancer cells to androgen-deprivation therapy in vitro and in vivo by reducing MRP levels and OXPHOS activity [140]. SAMMSON modulates the RNA–protein complex formation in both cytosol and mitochondria, thereby promoting a balanced enhancement of rRNA processing and protein synthesis in these cellular compartments, a phenomenon which facilitates cancer cell growth [141]. Inhibition of SAMMSON using an antisense oligonucleotide induces apoptosis and suppresses tumor growth in uveal melanoma patient-derived xenograft (PDX) models [142]. Disrupting the equilibrium through the inhibition of the mitoribosome biogenesis machinery is emerging as a promising approach in anticancer therapy.

The successful use of antibiotic agents targeting bacterial ribosomes highlights the potential for mitoribosome inhibitor use in cancer treatments. Tetracycline analogs, such as tigecycline, doxycycline, and COL-3, inhibit mitochondrial protein synthesis by binding to the mtSSU, leading to reduced OXPHOS activity and to the suppression of cancer cell proliferation [143,144,145]. Tigecycline has demonstrated anticancer efficacy across various cancer types, including AML, HCC, lymphoma, gastric, ovarian, and breast cancers [139,146]. Notably, it has been observed to synergize with chemotherapy and other agents, such as pyrvinium pamoate, enhancing its therapeutic potential [143,147,148]. In vivo studies have shown that treatment with tigecycline or doxycycline can effectively inhibit the metastasis of OSCC [149]. These findings suggest potential therapeutic benefits of these agents in preventing the spread of OSCC and improving patient outcomes. However, the translation from preclinical experiments into practical applications presents substantial challenges [139]. For example, a Phase 1 clinical trial assessing tigecycline as a single agent in AML patients demonstrated no significant pharmacodynamic changes or clinical responses [150]. This suggests that achieving meaningful therapeutic effects with a single agent may be particularly difficult in AML patients, highlighting the need for alternative strategies such as combination therapies. Additionally, short-term pre-operative treatments with doxycycline have been shown to significantly reduce the number of cancer stem cells in breast cancer patients [151], further underscoring the promise and complexity of these therapeutic approaches.

## 6. Conclusions

Well-functioning mitochondria can support the proliferation and energy metabolism of normal cells. These MRPs are necessary for the composition of mitoribosomes, with or without a role in the assembly and translation of mitochondrial DNA. The distinct expression patterns of certain MRPs in relation to different cancers suggest their potential as diagnostic and prognostic biomarkers for these diseases. However, further studies are warranted to validate these findings. Targeting MRPs and mitoribosome biogenesis is emerging as a promising approach in anticancer therapy. Nevertheless, translating this potential into clinical practice will require extensive research to establish its efficacy and safety. To date, the specific mechanisms that trigger cancer development and promote its progression remain largely unknown. Investigating the functional roles of these MRPs across different types of cancer could uncover key mechanisms driving disease development and progression, as well as revealing the extra-ribosomal properties. This, in turn, could provide valuable insights and clues for the development of innovative therapeutic strategies.

## Figures and Tables

**Table 1:** Differentially expressed MRPs across subtypes of breast cancer in TCGA.

Subtypes	Samples	Expression Pattern	Differentially Expressed MRPs *
Luminal A	563	Upregulation	MRPL12, MRPL14, MRPL24, MRPL41, MRPL55, MRPS12, MRPS30, MRPS34
Luminal B	210	Upregulation	MRPL12, MRPL13, MRPL14, MRPL15, MRPL17, MRPL21, MRPL24, MRPL27, MRPL47, MRPL55, MRPL58, CHCHD1, DAP3, MRPS12, MRPS23, MRPS28, MRPS30, MRPS34
Basal-like	190	Upregulation	GADD45GIP1, MRPL2, MRPL4, MRPL9, MRPL12, MRPL13, MRPL14, MRPL15, MRPL36, MRPL37, MRPL47, MRPL51, MRPL55, DAP3, MRPS12, MRPS21, MRPS34
HER2-enriched	79	Upregulation	MRPL12, MRPL13, MRPL14, MRPL27, MRPL41, MRPL47, MRPL58, CHCHD1, MRPS12, MRPS34
Normal-like	70	Upregulation	MRPL13

* |log2 (FC)|> 1, *p* value < 0.001.

**Table 2:** Differentially expressed MRPs across pan-cancer based on the GEPIA database.

Cancer	Expression Pattern	Differentially Expressed MRPs *
AML	Downregulation	MRPL2, MRPL4, MRPL10, MRPL11, MRPL12, MRPL13, MRPL14, MRPL16, MRPL17, MRPL18, MRPL19, MRPL20, MRPL21, MRPL22, MRPL27, MRPL28, MRPL30, MRPL32, MRPL35, MRPL36, MRPL37, MRPL39, MRPL40, MRPL41, MRPL42, MRPL46, MRPL47, MRPL48, MRPL49, MRPL50, MRPL51, MRPL52, MRPL55, GADD45GIP1, MRPS18A, MRPS18B, MRPS18C, MRPS2, MRPS6, MRPS7, MRPS9, MRPS10, MRPS12, MRPS14, MRPS15, MRPS16, MRPS17, MRPS21, MRPS22, MRPS23, MRPS24, MRPS28, DAP3, MRPS33, MRPS34, MRPS35, CHCHD1, AURKAIP1
Breast invasive carcinoma	Upregulation	MRPL3, MRPL13, MRPL14, MRPL17, MRPL24, MRPL42, MRPL47, MRPS30, MRPS23, DAP3, MRPS34, MRPS35
Glioblastoma multiforme	Upregulation	MRPL3, MRPL11, MRPL13, MRPL14, MRPL19, MRPL22, MRPL24, MRPL36, MRPL39, MRPL40, MRPL42, MRPL44, MRPL47, MRPL49, MRPL52, MRPL53, MRPL54, MRPS6, MRPS10, MRPS12, MRPS14, MRPS16, MRPS23, MRPS28, DAP3, MRPS33, MRPS35
	Downregulation	MRPL41
Head and neck squamous cell carcinoma	Upregulation	MRPL47
HCC	Upregulation	MRPL9, MRPL13, MRPL14, MRPL15, MRPL17, MRPL21, MRPL24, MRPL47, MRPL55, MRPS16, MRPS17, MRPS21, MRPS23, DAP3
Kidney renal clear cell carcinoma	Downregulation	MRPS25
Lower grade glioma	Upregulation	MRPL3, MRPL11, MRPL14, MRPL19, MRPL24, MRPL42, MRPS18B, MRPS14, MRPS16, MRPS23, MRPS28
	Downregulation	MRPL41
Ovarian serous cystadenocarcinoma	Upregulation	MRPL3, MRPL12, MRPL13, MRPL14, MRPL15, MRPL34, MRPL35, MRPL37, MRPL47, MRPL48, MRPS18A, MRPS11, MRPS12, MRPS15, MRPS16, MRPS35, AURKAIP1
Pancreatic adenocarcinoma	Upregulation	MRPL1, MRPL3, MRPL4, MRPL9, MRPL11, MRPL12, MRPL13, MRPL14, MRPL15, MRPL16, MRPL17, MRPL18, MRPL19, MRPL20, MRPL21, MRPL22, MRPL24, MRPL27, MRPL28, MRPL30, MRPL33, MRPL34, MRPL35, MRPL36, MRPL41, MRPL42, MRPL43, MRPL44, MRPL46, MRPL47, MRPL48, MRPL49, MRPL50, MRPL51, MRPL52, MRPL53, MRPL54, MRPL55, MRPL57, GADD45GIP1, MRPS30, MRPS18A, MRPS18C, MRPS5, MRPS6, MRPS7, MRPS10, MRPS11, MRPS12, MRPS14, MRPS15, MRPS16, MRPS17, MRPS21, MRPS23, MRPS27, MRPS28, DAP3, MRPS31, MRPS34, MRPS35, CHCHD1, AURKAIP1
Prostate adenocarcinoma	Upregulation	MRPL17
Thyroid cancer	Upregulation	MRPL14, MRPL17
	Downregulation	PTCD3
Uterine corpus endometrial carcinoma	Upregulation	MRPL3, MRPL12, MRPL13, MRPL14, MRPL15, MRPL17, MRPL19, MRPL47, MRPL51, MRPL52, GADD45GIP1, MRPS12, MRPS15, MRPS16, MRPS17, MRPS26, MRPS33, MRPS34, CHCHD1, AURKAIP1
	Downregulation	PTCD3
Uterine carcinosarcoma	Upregulation	MRPL3, MRPL11, MRPL12, MRPL13, MRPL14, MRPL15, MRPL17, MRPL47, MRPL51, MRPL53, MRPS6, MRPS12, MRPS15, MRPS16, MRPS34, CHCHD1, AURKAIP1
	Downregulation	PTCD3

* |log2 (FC)| > 1, q value < 0.01.
